# Screening of Particulate Matter Reduction Ability of 21 Indigenous Korean Evergreen Species for Indoor Use

**DOI:** 10.3390/ijerph18189803

**Published:** 2021-09-17

**Authors:** Bo-Kook Jang, Kyungtae Park, Sang Yeob Lee, Hamin Lee, Soo Ho Yeon, Boran Ji, Cheol Hee Lee, Ju-Sung Cho

**Affiliations:** 1 Division of Animal, Horticultural and Food Sciences, Chungbuk National University, Cheongju 28644, Korea; jangbk@chungbuk.ac.kr (B.-K.J.); pkt4418@naver.com (K.P.); lsylsyder@naver.com (S.Y.L.); hamin0627@naver.com (H.L.); darkcl@naver.com (S.H.Y.); jbr9743@naver.com (B.J.); leech@chungbuk.ac.kr (C.H.L.); 2 Brain Korea 21 Center for Bio-Health Industry, Chungbuk National University, Cheongju 28644, Korea

**Keywords:** aerosol particles, indoor air quality, indoor plants, PM_2.5_, PM_10_, relative humidity

## Abstract

The formation and pollution of particulate matter (PM), a side effect of rapid industrialization and urbanization, is considered a global issue. However, various plant species are able to effectively capture and reduce atmospheric PM concentrations. We investigated the indoor growth and morphology of 21 indigenous Korean evergreen species at low light intensities to ascertain their ability to reduce PM of aerosol particles in a closed acrylic chamber. The decrease in PM mass concentration differed significantly across species, with a significant correlation (8 h; *p* < 0.001). The reduction in the mass concentration of PM differed with particle size and across species. The highest reduction of PM_2.5_ occurred after 8 h with *Dryopteris lacera* (86.8%), *Ilex × wandoensis* (84.9%), *Machilus thunbergii* (84.3%), and *Rhododendron brachycarpum* (84.0%). Reduction of PM_10_ after 8 h was highest with *Cephalotaxus harringtonii* (98.3%), *I. × wandoensis* (98.5%), *M. thunbergii* (98.5%), and *R. brachycarpum* (98.3%). Plant morphological characteristics (category, plant height, leaf shape, leaf area) and relative humidity were closely related to the decrease in PM mass concentration. In conclusion, our findings can be used to identify Korean plant species that can reduce PM concentration and are suitable for indoor use.

## 1. Introduction

Particulate matter (PM) produced by industrialization and urbanization is a source of air pollution and poses a direct risk to ecosystems and human health. The formation of PM is related to natural or anthropogenic factors such as aeolian erosion, ash, heavy metals, pollen, industrialization, fossil fuel combustion, and vehicle emissions [1,2]. The generally used particle sizes of PMs are <2.5 and <10 μm, classified as PM_2.5_ and PM_10_, respectively. Very small PM suspended in the air can easily be inhaled and accumulate in the human body during respiration and is a direct cause of respiratory, cardiovascular, and pulmonary diseases [3,4,5]. Furthermore, as PM pollution has a significant impact on human health and social activities, many researchers are focusing on ways to remove PM and improve air quality. 

Plants have been shown to effectively accumulate and reduce PM from the atmosphere. This ability depends primarily on leaf characteristics, with significant differences exhibited between plant species [6,7]. In particular, specific structural characteristics, such as the presence of cuticles and wax layers, allow leaf surfaces to effectively capture PM [8]. The ability to capture PM is affected by a number of factors including plant size, leaf type, leaf arrangement, leaf microstructure, leaf surface roughness, leaf area, rainfall, temperature, and humidity [9,10,11,12,13,14,15]. By planting or placing plants indoors in living spaces, PM can be efficiently removed and air quality improved. However, as most research on the reduction of PM by plants has focused on the outdoor environment, there is a need for research on the potential of indoor plants to reduce PM [13,14,15]. 

At a time when social activity is limited by the COVID-19 outbreak, the reduction of pollutants through the presence of indoor plants can improve both air quality and public mental health [16]. However, indoor environments generally have a lower light intensity than outdoor environments and, depending on the space, exposure to continuous shade or excessive light can adversely affect plant growth [17,18]. Therefore, suitable light conditions are essential for indoor plants. Within this context, we investigate the PM reduction effect of plant species capable of growing indoors in low light conditions, using an enclosed acrylic chamber. Our findings provide important information on the PM reduction ability of various plant species native to Korea. 

## 2. Materials and Methods

### 2.1. Plant Material

Seedlings of 21 different plant species were purchased from various regions in South Korea in 2019 (Table 1). Young seedlings were transplanted into 15 cm pots containing artificial soil, with a 2:1 (*v/v*) ratio of horticultural substrate (Hanareum no. 2; Shinsung Mineral Co., Ltd., Goesan, Korea) and decomposed granite (4 mm; Samgye Masato, Gimhae, Korea). Thereafter, plants were kept in a glass greenhouse and used for PM-related experiments. Data obtained from the experiments included the number of plants and specific growth parameters used for investigating the reduction of PM (Table 1), as well as leaf shape (Figure S1).

### 2.2. Plant Growth and Chlorophyll Fluorescence Analysis 

Seedling growth and chlorophyll fluorescence parameters at various light intensities were used to investigate the potential of 21 plant species for indoor use. This study was carried out in a growth room blocked from external light [18]. Seedlings were grown in a multi-stage type experimental bed at different light intensities (white LED, HT100-5700, BISSOL LED, Seoul, Korea) of 10, 50, 100, and 200 μmol·m^−2^·s^−1^ (PPFD). All experiments were completely randomized, with four plants of each species grown for 8 or 10 weeks per light intensity level. Control plants were grown during the same period in a glass greenhouse at Chungbuk National University. The light intensity in the glass greenhouse, measured at noon in July, ranged from 170.4 to 399.2 PPFD. Growth responses were evaluated using the following parameters: plant height, number of leaves, leaf length, leaf width, and chlorophyll content (Minolta SPAD-502 chlorophyll meter, Minolta Camera Co., Ltd., Osaka, Japan). Chlorophyll fluorescence was analyzed with a portable PAM fluorometer (FP-110, Photon Systems Instruments, Drásov, Czech Republic). After growing, leaves were dark-adapted for 30 min using a leaf-clip, and were then measured three times. Chlorophyll fluorescence parameters were estimated using JIP-test with the following parameters: maximum quantum yield of PS II (Fv/Fm), performance index on absorption basis (Pi_Abs), electron transport per reaction center (ETo/RC), and dissipation per reaction center (DIo/RC). The growth room was maintained at a 12 h/12 h photoperiod at a temperature of 25 ± 1 °C and a humidity of 55 ± 3%. The same irrigation frequency (once every three days) was used in both the growing room and the greenhouse.

### 2.3. Particulate Matter (PM) Reduction Experiment

**Figure 1:**
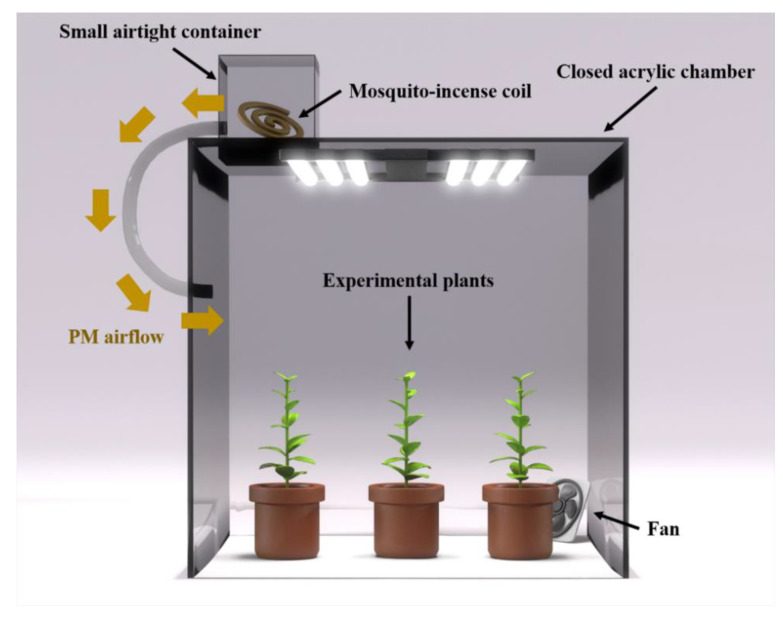
The design schematic of the closed acrylic chamber used for the particle matter (PM) reduction tests.

To investigate the reduction in PM, a closed acrylic chamber (800mm × 800mm × 1000 mm) was manufactured and used for experimentation (Figure 1). A fan (2300 RPM, 26.9 CFM, Dong Ke Technology, Taitung City, Taiwan) was placed inside the chamber and a small airtight container was used to add incense PM. The light intensity source of the chamber was set at a distance of 50 cm from the floor to ensure a luminous intensity (100 PPFD white-LED). In total, three chambers were manufactured and used for experimentation under identical conditions. The mean mass PM_2.5_ and PM_10_ concentration of the empty chamber (zero) before incense PM injection for 8 h was 24.1–37.7 μg·m^−3^ (*n* = 3).

### 2.4. Particulate Matter (PM) Reduction Effect of Plant Species

Prior to the experiment, the inner sides of the chamber were cleaned with dust-free papers and an anti-static agent (F-150, Nambang CNA Co., Ltd., Pyeongtaek, Korea) was used 6 h prior to the experiment to eliminate the effect of electrostatic force. After placing the seedlings inside the chamber, it was closed and a mosquito-incense coil (Happy Home mosquito-incense coil; Yuhan Yanghang, Seoul, Korea) was lit and allowed to burn in a small airtight container for 10 min, with the smoke channeled into the chamber through a valve. After the supply of incense PM into the chamber, the fan inside the chamber was turned on for 3 min to evenly distribute the incense PM. The fan was switched off, the mass concentration was controlled to 900–1000 μg·m^−3^ (PM_2.5_) and 1000–1100 μg·m^−3^ (PM_10_), after which the valve was closed. The incense PM concentration and relative humidity (RH) in the chamber were measured every hour up to 8 h. Mass concentration value of the empty chamber without plants was measured and used as a blank for comparison with the mass concentration in the chamber with plant species. We also compared changes in mass concentration by placing four artificial plants (Green Eucalyptus, 1015613, AsungDaiso, Seoul, Korea) in the chamber instead of plant species. The artificial plant information used in the experiment was as follows: plant height (49.8 cm), number of leaves per plant (25 ea), leaf area (18.2 cm^2^), leaf area per plant (455.6 cm^2^), leaf area per chamber (1822.5 cm^2^).

### 2.5. Data Collection and Statistical Analysis

**Figure 2:**
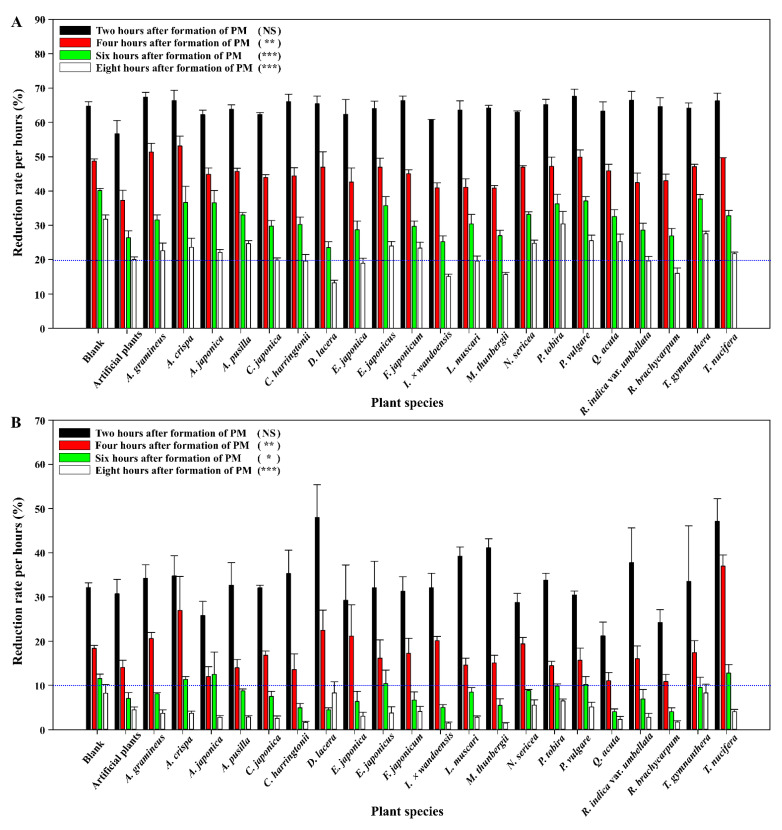
Reduction effect of plant species on mass concentration of (**A**) PM_2.5_ and (**B**) PM_10_. Vertical bars represent mean ± SE (*n* = 3). Reduction of particle matter (PM) mass concentration by plant species was analyzed using a one-way ANOVA. NS, not significant; *, **, and ***, significance at *p* < 0.05, 0.01, and 0.001, respectively.

Plant height, number of leaves, leaf length, leaf width, leaf area, and chlorophyll content of each plant were investigated before the experiment (*n* = 9). After the experiment, all leaves were collected and the leaf area was measured using a leaf area meter (Li-3100, LI-COR Inc., Lincoln, NE, USA) (*n* = 9). Incense PM was detected with a particle counter (Aerocet-531S, Met One Instruments Inc., Grants Pass, OR, USA) and quantified in units of mass concentration (μg·m^−3^) in terms of PM_2.5_ (<2.5 μm), PM_10_ (<10 μm), and total suspended particulate (TSP) (*n* = 3). The PM_2.5_ and PM_10_ data were used to convert the initial value of the controlled mass concentration to 100%, and all the values of the mass concentration measured afterwards were also expressed in percentage (Figure 2). Moreover, because the measured mass concentration of PM_10_ includes PM_2.5_, the PM_10_ concentration excluding PM_2.5_ was calculated. Data were calculated as mean ± standard error for all parameters in each treatment, and a factorial analysis was performed using Duncan’s multiple range test with a significance level of *p* < 0.05 using SAS software (Version 9.4, SAS Institute Inc., Cary, NC, USA). The reduction in PM was analyzed using one-way analysis of variance (ANOVA) with SAS. Principal component analysis (PCA) was performed using R software (Version 4.1.0, R Foundation for Statistical Computing, Vienna, Austria) to investigate the correlation between PM reduction and each parameter. The parameters used for PCA included plant category (CTGY), leaf shape (LS), time taken to reach ≥90% relative humidity (RH), plant height (PH), number of leaves (NL), leaf area (LAL), leaf area per plant (LAPP), total leaf area of plants in chamber (TLA), mass concentration of reduced PM_2.5_ after 8 h (<PM_2.5_), mass concentration of reduced PM_10_ after 8 h (<PM_10_), and mass concentration of reduced TSP after 8 h (TSP).

## 3. Results and Discussion

### 3.1. Effect of Plant Species on Reduction of PM_2.5_ and PM_10_

In this study, we evaluated the PM reduction ability and indoor ornamental plant potential of 21 indigenous species. All selected species grew sufficiently, even in low light conditions of 100 PPFD, based on growth and chlorophyll fluorescence parameters (Tables S1 and S2) [18]. Although the mass concentration of PM in the chamber generally decreased over time, we recorded a significant difference in the reduction concentration across the various plant species (Figure 2). The mass concentration of PM_2.5_ was reduced by more than 30% in 2 h, 50% after 4 h, 60% after 6 h, and more than 70% after 8 h in all species (Figure 2A). The highest reduction of the PM_2.5_ concentration after 8 h was recorded with *Dryopteris lacera* (86.8%), *Ilex × wandoensis* (84.9%), *Machilus thunbergii* (84.3%), and *Rhododendron brachycarpum* (84.0%). The decrease in PM_2.5_ mass concentration was correlated with plant species, especially after 6 h (*p* < 0.001). The mosquito-incense coil used in this study produces unhealthy pollutants when burned, primarily as fine particles of <2.5 μm [19]. Therefore, the mass concentration of suspended matter in the chamber consisted of predominantly PM_2.5_, which has a significantly smaller diameter than PM_10_. As the mass concentration of PM_10_ is affected by gravitational sedimentation, it is estimated that the sedimentation of PM_2.5_ decreases by 1/16 due to gravity [13,20]. This difference suggests that the reduction rate of PM_10_ suspended matter is higher than that of PM_2.5_ [13]. In our study, PM_10_ was reduced by more than 40% in 2 h, which was at a much faster rate than that of PM_2.5_ (Figure 2B). The mass concentration of PM_10_ decreased by more than 96.4% across all plant species after 8 h, with *Cephalotaxus harringtonii* (98.3%), *I. × wandoensis* (98.5%), *M. thunbergii* (98.5%), and *R. brachycarpum* (98.3%) showing the highest rates of reduction. The decrease in PM_10_ mass concentration was correlated with plant species, after 8 h (*p* < 0.001). A difference in the reduction of suspended matter was observed according to the plant species and PM size. Among the 21 investigated species, *I. × wandoensis*, *M. thunbergii*, and *R. brachycarpum* were most effective in reducing suspended matter in both PM_2.5_ and PM_10_. Conversely, a similar decreasing trend was also investigated in the empty chamber without plants (blank). Thus, a difference in the reduction rate was evidently confirmed in both types of chambers (with and without plants). PM reduction by plants is assumed to be influenced not only by the effects of gravitational sedimentation, but also by the physiological functions of plants. Further, this was confirmed using artificial plants with suppressed physiological functions. Mechanisms of PM removal by plants are reported to include stomata uptake and deposition on leaf surface [21,22]. PM can block or pass through the stomata of plants and accumulate under the epidermis or in the palisade layer of leaves [22]. PM containing heavy metals can move throughout the plant through symplastic or apoplastic pathways [23]. Artificial plants showed a similar PM reduction trend to some plant species, but they showed a relatively low reduction rate compared to *I. × wandoensis*, *M. thunbergii*, and *R. brachycarpum* which were the most effective species for PM reduction. This is presumed to be because of the PM removal capacity of plants, and not because of gravitational sedimentation [21]. Although these results provide substantial evidence for PM removal by the physiological functions of plants, further studies are needed to comprehensively elucidate other mechanisms of PM removal.

### 3.2. Effect of Various Factors on Reduction of PM

We investigated the effect of various factors on PM_2.5_ and PM_10_ reduction including plant category, leaf shape, relative humidity, and leaf area (Table 2). All study species can be described as woody with elliptic leaves. In general, the larger the leaf surface area, the more efficient the reduction of PM [8,24]. It is well-known from several studies that the PM capture ability of plants varies widely across species and is closely related to leaf microstructure, leaf surface roughness, and leaf area [6,10,11,12].

**Figure 3:**
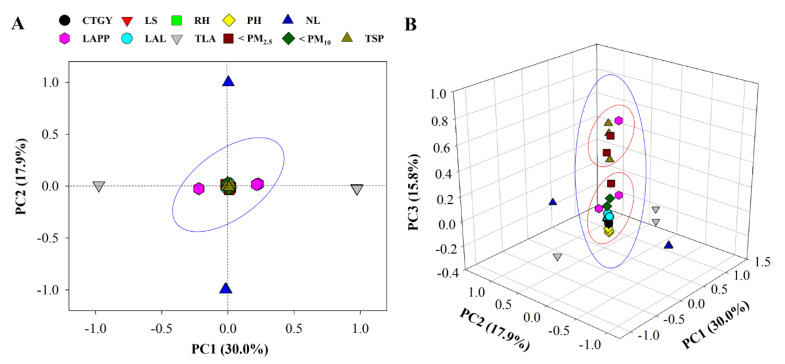
Principal component analysis (PCA) of various factors and particulate matter (PM) reduction by plants including (**A**) 3D plot of PCA and (**B**) 2D plot of PCA. Black represents categories of plants (CTGY), red represents leaf shape (LS), green represents the time to achieve ≥90% relative humidity (RH) in the acrylic chamber, yellow represents plant height (PH), blue represents the number of leaves (NL), pink represents the leaf area per plant (LAPP), see blue represents leaf area (LAL), gray represents the leaf area of total plants in chamber (TLA), brown represents the mass concentration of reduced PM_2.5_ after 8 h (<PM_2.5_), dark green represents the mass concentration of reduced PM_10_ after 8 h (<PM_10_), and deep yellow represents the mass concentration of reduced TSP after 8 h (TSP).

The PCA results revealed that various factors contributed to decreases in the mass concentration of suspended matter (Figure 3). In the two-dimensional PCA, all factors with the exception of TLA and NL formed clusters with PC1 at 30.0% and PC2 at 17.9%, representing 47.9% of the cumulative variance (Figure 3A). Results from the three-dimensional PCA increased explanatory power with PC3 (15.8%) and represented 63.7% of the cumulative variance (Figure 3B). In addition, in the 3D PCA plot, results showed two clusters between factors. Among the suspended matter in the chamber, PM_2.5_ was dominant and formed a cluster with TSP. The other factors CTGY, LS, RH, PH, LAPP, and LAL formed clusters with PM_10_. These results suggest that morphological characteristics of plant species are important factors affecting the reduction of PM mass concentration. In terms of PM accumulation, leaf characteristics such as leaf shape, leaf arrangement, and the presence of a wax layer and hair structure on the leaf surface are important factors influencing PM accumulation [6,8,14]. However, the reduction of PM in the chamber focuses on factors involved in gravitational sedimentation [13], one of which is the leaf area occupying the volume and area. In addition, our results indicate that the PM reduction rate increased as the RH in the chamber became rapidly saturated. Notably, plant species with high PM reduction rates reached an RH of 90% or higher between 60 and 100 min. These results are closely related to the hygroscopic and wetting mechanisms of fine particles [24], and indirectly suggest that the mass concentration of suspended matter is affected by RH [7,25]. The rapid increase in RH was due to evapotranspiration and is a physiological function of the leaf area and stomata of the plant. Ryu et al. [13] reported that the increase in RH in the chamber due to evapotranspiration from *Epipremnum aureum* improved the removal of PM smoke particles. Furthermore, it has also been reported that this increase in RH affects the deposition velocity of fine particles [26,27,28,29]. Therefore, leaf area and RH influenced the reduction of PM in the chamber. However, we acknowledge that we did not measure the amount of evapotranspiration in the study plants and therefore, cannot confirm the PM reduction at various RH levels. We suggest that this be considered in future studies.

## 4. Conclusions

In summary, the indigenous Korean evergreen plants used in this study were able to grow indoors under low light conditions, and had a significant PM reduction effect. Plant species showed a significant correlation with PM reduction. *I. × wandoensis*, *M. thunbergii*, and *R. brachycarpum* were most effective at PM reduction, with the rate of reduction differing according to the PM size. In general, PM_10_ was reduced at a faster rate than PM_2.5_. Analyses of the relationship between PM reduction, plant leaf characteristics, and RH showed that leaf area and RH had a significant effect on PM reduction. These results suggest that the increased RH related to evapotranspiration by plants in the chamber is involved in PM reduction. Thus, our study provides information on various plant species of Korea that can grow indoors and are effective at reducing PM.

## Figures and Tables

**Table 1:** Plant information and growth parameters of 21 species indigenous to Korea.

Species	Collection Region	No. of Plants/Chamber	Plant Height (cm)	No. of Leaves/Plant	Leaf Length (cm)	Leaf Width (cm)	Leaf Area (cm^2^)	Chlorophyll Content
*Acorus gramineus* Ation	Andong	5	17.5 ± 0.86	92.9 ± 8.12	20.9 ± 0.92	0.4 ± 0.02	3.6 ± 0.03	57.9 ± 2.08
*Ardisia crispa* (Thunb.) A.DC.	Seogwipo	5	24.3 ± 1.49	93.2 ± 11.50	7.6 ± 0.23	1.9 ± 0.08	4.6 ± 0.28	38.2 ± 1.58
*Ardisia japonica* (Thunb.) Blume	Seogwipo	8	11.4 ± 1.35	38.7 ± 5.17	5.2 ± 0.39	2.5 ± 0.12	5.1 ± 1.04	41.3 ± 1.40
*Ardisia pusilla* A.DC.	Seogwipo	5	6.3 ± 0.67	123.8 ± 16.99	2.5 ± 0.07	1.6 ± 0.06	77.1 ± 12.46	38.6 ± 1.52
*Camellia japonica* L.	Gangjin	4	52.9 ± 2.68	39.7 ± 3.36	7.4 ± 0.17	4.1 ± 0.16	15.1 ± 1.40	67.3 ± 2.14
*Cephalotaxus harringtonii* (Knight ex J.Forbes) K.Koch	Naju	5	21.2 ± 1.33	541.9 ± 39.82	3.8 ± 0.26	0.3 ± 0.01	0.5 ± 0.07	54.5 ± 1.95
*Dryopteris lacera* (Thunb.) Kuntze	Cheongju	4	17.5 ± 1.15	22.3 ± 1.43	26.6 ± 0.36	10.3 ± 0.30	57.1 ± 1.04	46.1 ± 1.13
*Eriobotrya japonica* (Thunb.) Lindl.	Gangjin	5	53.3 ± 3.47	17.3 ± 1.71	16.9 ± 0.88	5.5 ± 0.21	57.5 ± 9.70	44.5 ± 0.88
*Euonymus japonicus* Thunb.	Gangjin	5	50.7 ± 1.44	54.7 ± 5.10	4.1 ± 0.16	2.2 ± 0.13	5.2 ± 0.04	68.4 ± 1.76
*Farfugium japonicum* (L.) Kitam.	Seogwipo	5	17.1 ± 1.11	5.1 ± 0.56	6.7 ± 0.40	11.1 ± 0.75	73.5 ± 5.79	41.7 ± 1.30
*Ilex × wandoensis* C.F.Mill. & M.Kim	Wando	5	22.6 ± 0.96	54.6 ± 5.70	6.4 ± 0.18	2.9 ± 0.09	10.4 ± 0.43	57.8 ± 1.14
*Liriope muscari* (Decne.) L.H.Bailey	Andong	5	20.5 ± 0.84	96.2 ± 5.05	21.3 ± 0.56	0.4 ± 0.02	1.6 ± 0.11	62.4 ± 1.30
*Machilus thunbergii* Siebold & Zucc.	Gangjin	5	52.0 ± 1.64	38.9 ± 3.51	9.1 ± 0.43	3.6 ± 0.21	18.9 ± 0.76	41.0 ± 0.94
*Neolitsea sericea* (Blume) Koidz.	Wando	4	50.1 ± 2.55	36.6 ± 4.39	10.4 ± 0.29	4.2 ± 0.21	13.6 ± 1.16	41.1 ± 0.92
*Pittosporum tobira* (Thunb.) W.T.Aiton	Gangjin	5	37.2 ± 1.39	59.0 ± 8.12	5.0 ± 0.25	2.2 ± 0.09	7.0 ± 1.26	49.3 ± 1.65
*Polypodium vulgare* L.	Cheongju	5	7.3 ± 0.72	10.1 ± 1.35	11.7 ± 0.38	5.3 ± 0.24	11.2 ± 0.26	30.8 ± 2.11
*Quercus acuta* Thunb.	Wando	5	29.7 ± 1.54	31.2 ± 2.03	9.1 ± 0.51	2.9 ± 0.23	11.9 ± 0.55	36.4 ± 1.21
*Rhaphiolepis indica* var. *umbellata* (Thunb.) H.Ohashi.	Gangjin	5	59.6 ± 1.92	56.2 ± 4.78	8.1 ± 0.45	2.7 ± 0.10	9.5 ± 1.19	61.9 ± 1.97
*Rhododendron brachycarpum* D.Don ex G.Don	Seogwipo	4	22.2 ± 0.94	45.4 ± 4.80	10.7 ± 0.53	3.6 ± 0.13	15.2 ± 1.00	49.0 ± 1.27
*Ternstroemia gymnanthera* (Wight & Arn.) Sprague	Wando	4	28.1 ± 2.93	30.8 ± 4.85	8.8 ± 2.12	4.8 ± 2.08	6.2 ± 0.47	37.6 ± 2.33
*Torreya nucifera* (L.) Siebold & Zucc.	Naju	5	34.7 ± 1.46	1303.4 ± 68.92	2.3 ± 0.09	0.3 ± 0.01	0.3 ± 0.01	67.1 ± 1.79

**Table 2:** Morphological characteristics of 21 plant species investigated regarding the reduction in mass concentration of particulate matter (PM).

Species	Category	Leaf Shape	Leaf Area/ Plant (cm^2^)	Leaf Area/ Chamber (cm^2^)	≥90% RH ^2^ (time)	PM_2.5_ Reduction after 8 h (%)	PM_10_ Reduction after 8 h (%)
*Acorus gramineus*	Herb	Linear	335.5 ± 27.66 hi ^1^	1677.5 ± 138.28 e–h	100	77.4 ± 2.21 c–f	96.3 ± 0.80 a–d
*Ardisia crispa*	Shrub	Lanceolate	432.6 ± 50.52 f–h	2162.8 ± 252.59 c–g	140	76.4 ± 2.64 c–f	96.3 ± 0.47 a–d
*Ardisia japonica*	Shrub	Ovate	195.7 ± 38.34 jk	1565.4 ± 306.75 f–h	120	77.9 ± 0.87 c–e	97.2 ± 0.32 a–c
*Ardisia pusilla*	Subshrub	Elliptic	164.8 ± 22.43 jk	824.2 ± 112.16 ij	180	75.3 ± 0.88 d–f	97.1 ± 0.30 a–c
*Camellia japonica*	Subtree	Ovate	588.2 ± 19.55 de	2352.9 ± 78.18 c–e	180	80.1 ± 0.63 b–d	97.4 ± 0.50 a–c
*Cephalotaxus harringtonii*	Shrub	Linear	244.1 ± 17.24 i–k	1220.4 ± 86.20 h–j	80	80.4 ± 1.89 b–d	98.3 ± 0.23 ab
*Dryopteris lacera*	Herb/ferns	pinnate	1274.4 ± 48.10 a	5097.7 ± 192.41 a	60	86.8 ± 0.82 a	91.7 ± 2.54 e
*Eriobotrya japonica*	Subtree	Elliptic	989.4 ± 144.89 b	4947.1 ± 724.44 a	60	81.0 ± 1.51 bc	96.9 ± 0.88 a–c
*Euonymus japonicus*	Shrub	Elliptic	284.8 ± 37.52 ij	1423.9 ± 187.58 g–i	120	76.0 ± 1.32 c–f	96.2 ± 1.33 a–d
*Farfugium japonicum*	Herb	Reniform	366.7 ± 27.08 g–i	1833.7 ± 135.41 e–h	120	76.7 ± 1.71 c–f	95.9 ± 1.06 a–d
*Ilex × wandoensis*	Shrub	Oblong	567.4 ± 37.88 d–f	2836.9 ± 189.39 c	60	84.9 ± 0.67 ab	98.5 ± 0.28 a
*Liriope muscari*	Herb	Linear	156.9 ± 12.97 jk	784.5 ± 64.84 ij	120	80.5 ± 1.52 b–d	97.2 ± 0.25 a–c
*Machilus thunbergii*	Tree	Elliptic	731.6 ± 28.20 c	3658.0 ± 140.98 b	60	84.3 ± 0.54 ab	98.5 ± 0.12 a
*Neolitsea sericea*	Tree	Elliptic	486.3 ± 32.25 e–g	1945.0 ± 129.01 d–h	220	75.2 ± 0.90 d–f	94.5 ± 1.20 c–e
*Pittosporum tobira*	Shrub	Obovate	388.0 ± 28.64 g–i	1940.0 ± 143.21 d–h	160	69.6 ± 3.58 g	93.5 ± 0.43 de
*Polypodium vulgare*	Herb/ferns	pinnate	112.6 ± 2.24 k	562.8 ± 11.21 j	180	74.5 ± 1.64 ef	94.9 ± 1.10 b–d
*Quercus acuta*	Tree	Ovate	369.2 ± 6.47 g–i	1846.0 ± 32.35 e–h	120	74.8 ± 2.24 ef	97.6 ± 0.64 a–c
*Rhaphiolepis indica* var. *umbellata*	Shrub	Obovate	532.1 ± 59.65 ef	2660.6 ± 298.23 cd	80	80.4 ± 1.33 b–d	97.2 ± 0.92 a–c
*Rhododendron brachycarpum*	Shrub	Elliptic	679.5 ± 41.53 cd	2717.8 ± 166.11 c	100	84.0 ± 1.55 ab	98.3 ± 0.34 ab
*Ternstroemia gymnanthera*	Subtree	Elliptic	191.6 ± 15.97 jk	766.3 ± 63.90 ij	240	72.5 ± 0.81 fg	91.7 ± 1.98 e
*Torreya nucifera*	Tree	Linear	448.9 ± 26.99 e–h	2244.4 ± 134.95 c–f	60	78.3 ± 0.46 c–e	95.9 ± 0.47 a–d

^1^ Different letters indicate a significant difference using Duncan’s multiple range test at *p* < 0.05. ^2^ Time taken to reach ≥90% RH.

## Data Availability

Not applicable.

## Supplementary Materials

The following are available online at https://www.mdpi.com/article/10.3390/ijerph18189803/s1: Figure S1 Leaf shape of plant species and artificial plant used in this study. Table S1 Effect of light intensity on the growth parameters of 19 plant species. Table S2 Effect of light intensity on chlorophyll fluorescence parameters of 19 plant species.
