# Supplementary material for: Screening of Particulate Matter Reduction Ability of 21 Indigenous Korean Evergreen Species for Indoor Use
Source: Int J Environ Res Public Health. 2021 Sep 17;18(18):9803. doi: 10.3390/ijerph18189803 (PMC8466017; doi:10.3390/ijerph18189803)
Supplement: Supplementary file 1 [file ijerph-18-09803-s001.zip › ijerph-1322198-supplementary.pdf]

International Journal of
Environmental Research and Public Health

MDPI

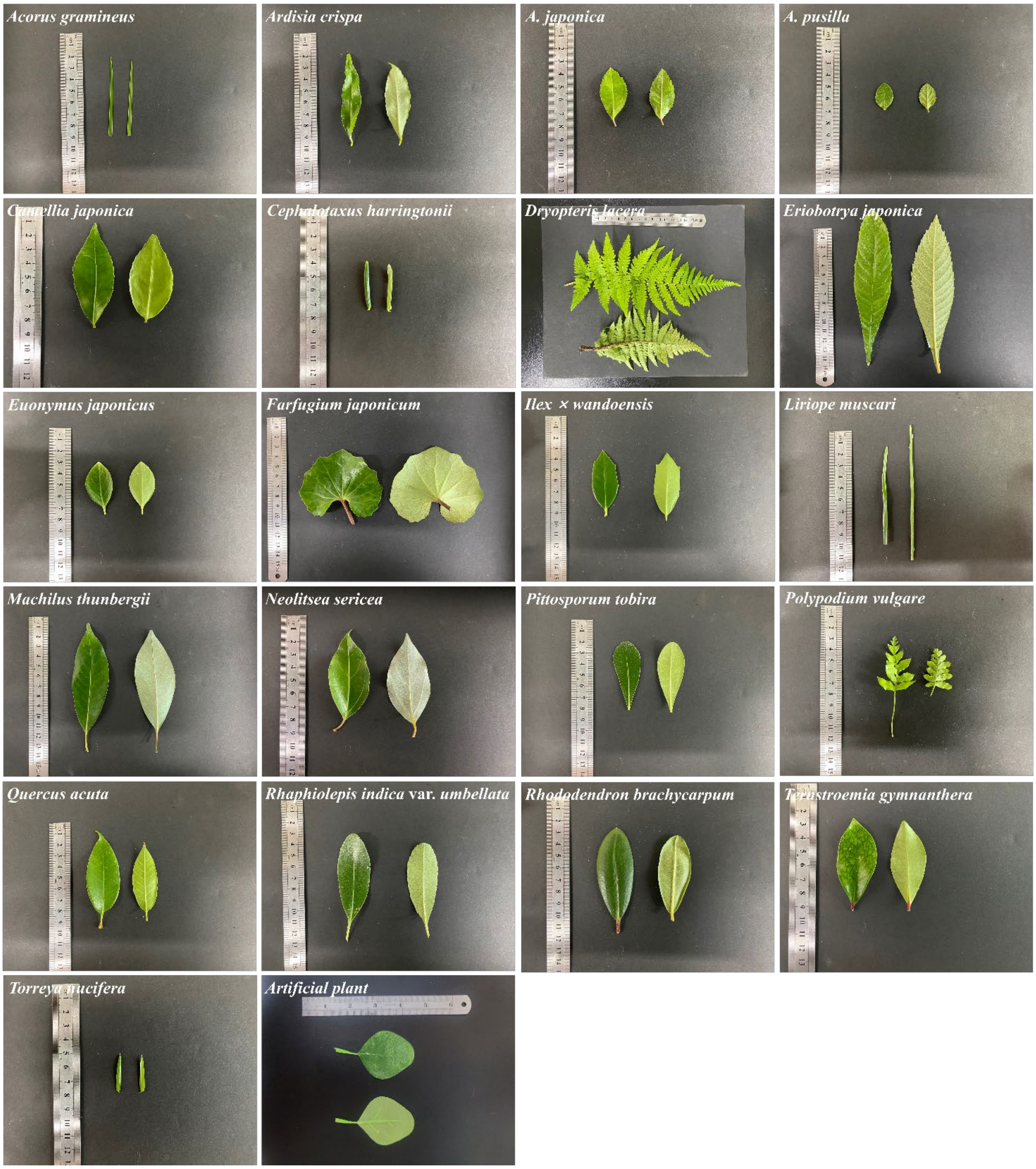

Figure S1. Leaf shape of plant species and artificial plant used in this study.

Table S1. Effect of light intensity on the growth parameters of 19 plant species.

Species	Light intensity (PPFD)	Plant height (cm)	Leaf number (ea/plant)	Leaf length (cm)	Leaf width (cm)	Chlorophyll content
Ilex × wandoensis	Control[1]	21.2 ± 1.76 a[2]	41.3 ± 6.64 a	6.8 ± 0.80 a	3.5 ± 0.13 a	47.7 ± 3.92 c
	10	21.4 ± 1.97 a	24.8 ± 1.49 b	7.3 ± 0.41 a	3.3 ± 0.10 a	70.9 ± 2.91 a
	50	22.9 ± 1.04 a	26.5 ± 2.25 b	6.0 ± 0.39 a	3.2 ± 0.24 a	63.6 ± 0.35 b
	100	20.0 ± 1.04 a	29.3 ± 2.87 b	7.4 ± 0.51 a	3.4 ± 0.37 a	60.1 ± 1.26 b
	200	20.1 ± 1.09 a	27.5 ± 3.52 b	6.5 ± 0.30 a	3.2 ± 0.32 a	63.2 ± 1.96 b
Euonymus japonicus	Control	40.0 ± 2.49 b	160.7 ± 19.23 a	4.8 ± 0.22 b	2.7 ± 0.13 ab	69.4 ± 0.88 a
	10	48.3 ± 2.83 ab	187.3 ± 4.26 a	4.8 ± 0.23 b	2.1 ± 0.12 b	74.4 ± 0.96 a
	50	46.3 ± 4.15 ab	184.0 ± 7.23 a	4.8 ± 0.06 b	2.3 ± 0.10 b	71.6 ± 2.10 a
	100	50.0 ± 3.48 a	187.8 ± 5.65 a	4.7 ± 0.26 b	2.3 ± 0.14 b	62.6 ± 2.26 b
	200	53.9 ± 1.18 a	223.8 ± 8.43 a	5.5 ± 0.08 a	3.3 ± 0.63 a	59.7 ± 2.30 b
Cephalotaxus harringtonii	Control	19.9 ± 1.30 a	252.2 ± 15.78 a	2.6 ± 0.03 ab	0.3 ± 0.01 bc	60.3 ± 3.73 a
	10	22.8 ± 0.79 a	153.7 ± 15.03 c	2.7 ± 0.09 ab	0.3 ± 0.02 a	61.2 ± 1.82 a
	50	22.0 ± 0.80 a	219.2 ± 13.18 ab	2.5 ± 0.20 b	0.3 ± 0.00 ab	64.3 ± 2.84 a
	100	21.2 ± 1.21 a	202.6 ± 13.60 b	2.9 ± 0.12 a	0.3 ± 0.00 ab	65.1 ± 4.22 a
	200	21.6 ± 1.11 a	230.3 ± 11.90 ab	2.5 ± 0.09 b	0.2 ± 0.02 c	70.5 ± 3.65 a
Rhododendron brachycarpum	Control	23.0 ± 1.29 a	33.3 ± 4.98 c	13.4 ± 1.08 a	4.6 ± 0.35 a	52.8 ± 4.25 a
	10	22.6 ± 0.93 a	58.0 ± 2.65 b	9.0 ± 0.85 b	2.8 ± 0.06 b	45.5 ± 3.72 a
	50	23.6 ± 0.13 a	68.0 ± 7.00 b	8.8 ± 0.76 b	2.9 ± 0.09 b	52.1 ± 2.35 a
	100	23.1 ± 0.86 a	66.7 ± 3.84 b	8.5 ± 0.55 b	3.2 ± 0.12 b	49.5 ± 3.24 a
	200	20.7 ± 1.95 a	83.0 ± 4.58 a	8.8 ± 0.75 b	2.9 ± 0.25 b	52.3 ± 7.70 a
Quercus acuta	Control	30.4 ± 1.53 a	22.5 ± 2.97 a	9.0 ± 0.18 a	3.1 ± 0.13 a	37.0 ± 2.49 b
	10	22.0 ± 1.25 b	18.0 ± 1.08 a	8.6 ± 0.53 a	2.6 ± 0.19 b	42.6 ± 2.74 a
	50	23.3 ± 0.86 b	17.0 ± 0.58 a	8.7 ± 0.51 a	2.4 ± 0.12 b	42.4 ± 1.26 a
	100	24.0 ± 0.40 b	19.3 ± 1.86 a	8.7 ± 0.28 a	2.7 ± 0.06 b	35.6 ± 1.18 b
	200	24.1 ± 1.62 b	22.7 ± 3.18 a	8.7 ± 0.06 a	2.6 ± 0.19 b	34.0 ± 0.94 b
Neolitsea sericea	Control	37.8 ± 0.86 a	22.8 ± 2.02 a	10.1 ± 0.19 a	3.6 ± 0.09 b	36.6 ± 0.84 cd
	10	33.4 ± 2.32 b	25.0 ± 2.08 a	11.4 ± 0.77 a	4.2 ± 0.18 ab	46.7 ± 0.42 a
	50	39.1 ± 2.28 a	25.0 ± 1.68 a	11.3 ± 0.21 a	4.3 ± 0.28 a	43.4 ± 0.98 b
	100	38.3 ± 1.02 a	23.5 ± 1.19 a	11.3 ± 0.46 a	4.3 ± 0.16 ab	39.3 ± 0.91 c
	200	39.3 ± 1.13 a	24.5 ± 1.04 a	11.5 ± 0.14 a	4.0 ± 0.28 ab	34.7 ± 1.01 d
Machilus thunbergii	Control	36.7 ± 1.48 a	30.5 ± 3.92 a	9.8 ± 0.16 ab	3.6 ± 0.23 ab	33.0 ± 0.97 d
	10	29.1 ± 2.32 b	16.3 ± 1.44 b	9.9 ± 0.78 ab	4.3 ± 0.55 a	49.9 ± 1.22 ab
	50	27.6 ± 1.14 b	26.7 ± 2.96 ab	10.7 ± 0.56 a	4.0 ± 0.27 ab	52.4 ± 1.92 a
	100	29.2 ± 0.80 b	25.7 ± 0.88 ab	10.7 ± 0.22 a	4.0 ± 0.23 ab	47.2 ± 0.85 b
	200	25.7 ± 1.59 b	31.0 ± 5.82 a	9.0 ± 0.15 b	3.2 ± 0.21 b	39.7 ± 1.26 c
Ardisia crispa	Control	20.4 ± 0.53 ab	20.3 ± 5.21 a	9.0 ± 0.43 a	2.6 ± 0.10 a	41.7 ± 2.79 a-c
	10	20.8 ± 1.31 ab	6.3 ± 0.63 b	8.7 ± 0.74 a	3.2 ± 0.31 a	55.2 ± 6.21 a
	50	19.6 ± 2.78 b	10.0 ± 2.04 b	8.2 ± 0.20 a	2.8 ± 0.24 a	45.6 ± 5.41 ab
	100	21.8 ± 1.61 ab	13.0 ± 2.74 ab	9.4 ± 0.52 a	2.8 ± 0.09 a	32.7 ± 0.94 bc

	200	26.8 ± 2.63 a	11.3 ± 2.36 b	8.0 ± 0.42 a	2.6 ± 0.04 a	30.0 ± 2.61 c
A. pusilla	Control	7.0 ± 0.09 a	16.3 ± 2.91 b	3.4 ± 0.10 ab	2.4 ± 0.23 a	44.3 ± 2.37 ab
	10	8.1 ± 0.56 a	20.3 ± 2.58 ab	3.6 ± 0.29 ab	2.4 ± 0.08 a	45.3 ± 1.03 ab
	50	8.3 ± 1.33 a	27.5 ± 4.93 a	3.8 ± 0.26 a	2.5 ± 0.13 a	48.6 ± 2.52 ab
	100	9.8 ± 1.75 a	28.2 ± 3.22 a	3.5 ± 0.23 ab	2.4 ± 0.08 a	52.1 ± 3.15 a
	200	8.7 ± 1.06 a	19.3 ± 1.17 ab	2.9 ± 0.22 b	2.7 ± 0.18 a	41.3 ± 2.70 b
A. japonica	Control	12.2 ± 1.39 a	2.7 ± 0.67 a	5.7 ± 0.39 b	2.5 ± 0.17 a	38.2 ± 2.23 a
	10	14.9 ± 2.00 a	6.5 ± 2.18 a	7.3 ± 0.61 ab	3.2 ± 0.29 a	39.5 ± 1.78 a
	50	12.6 ± 1.23 a	7.3 ± 1.75 a	7.9 ± 0.25 a	3.5 ± 0.31 a	39.1 ± 0.74 a
	100	14.9 ± 0.70 a	6.7 ± 0.33 a	6.8 ± 0.34 ab	3.1 ± 0.06 a	38.9 ± 2.98 a
	200	13.1 ± 2.44 a	6.8 ± 1.89 a	6.8 ± 0.64 ab	3.0 ± 0.44 a	36.3 ± 2.61 a
Pittosporum tobira	Control	35.3 ± 3.64 a[y]	57.2 ± 6.73 a	5.6 ± 0.32 b	2.3 ± 0.11 ab	44.0 ± 1.49 b
	10	31.4 ± 2.25 a	58.7 ± 6.74 a	6.7 ± 0.32 a	2.5 ± 0.23 ab	53.4 ± 2.40 a
	50	33.4 ± 1.48 a	52.0 ± 1.53 a	6.8 ± 0.27 a	2.7 ± 0.03 a	57.5 ± 2.34 a
	100	36.9 ± 1.27 a	51.5 ± 6.25 a	7.0 ± 0.42 a	2.5 ± 0.14 ab	52.9 ± 3.54 a
	200	35.7 ± 1.33 a	53.3 ± 4.27 a	6.4 ± 0.18 ab	2.2 ± 0.10 b	53.5 ± 2.90 a
Rhaphiolepis indica var. umbellata	Control	40.1 ± 1.29 a	49.8 ± 4.11 b	9.5 ± 0.48 a	2.6 ± 0.16 a	46.8 ± 1.36 c
	10	37.6 ± 1.90 a	47.0 ± 5.90 b	7.0 ± 0.32 b	2.3 ± 0.05 a	65.0 ± 1.15 a
	50	38.3 ± 2.95 a	49.3 ± 5.85 b	8.0 ± 0.16 b	2.7 ± 0.18 a	69.1 ± 0.82 a
	100	43.7 ± 2.52 a	69.5 ± 3.07 a	8.0 ± 0.31 b	2.5 ± 0.15 a	54.0 ± 0.55 b
	200	43.5 ± 4.78 a	65.8 ± 4.82 a	8.1 ± 0.26 b	2.7 ± 0.13 a	56.0 ± 4.11 b
Eriobotrya japonica	Control	38.3 ± 1.96 a[x]	17.3 ± 2.02 a	12.2 ± 0.29 b	4.1 ± 0.12 b	44.6 ± 1.41 c
	10	32.8 ± 0.74 a	18.0 ± 1.35 a	14.7 ± 0.97 ab	4.8 ± 0.21 ab	56.2 ± 0.88 b
	50	33.2 ± 2.39 a	17.8 ± 1.80 a	15.0 ± 1.27 ab	5.5 ± 0.46 ab	55.4 ± 2.61 b
	100	38.2 ± 1.53 a	18.0 ± 0.58 a	16.2 ± 1.80 a	5.5 ± 0.71 ab	58.1 ± 2.29 b
	200	32.4 ± 2.10 a	18.0 ± 0.58 a	14.9 ± 1.29 ab	5.5 ± 0.54 a	63.9 ± 0.90 a
Torreya nucifera	Control	33.9 ± 1.26 a[y]	793.7 ± 57.15 a	2.6 ± 0.14 bc	0.3 ± 0.00 a	63.2 ± 2.76 b
	10	25.8 ± 1.46 b	715.5 ± 127.84 a	2.5 ± 0.11 c	0.3 ± 0.00 a	73.2 ± 2.28 a
	50	32.8 ± 1.82 a	1125.0 ± 71.47 a	2.7 ± 0.18 ab	0.3 ± 0.04 a	71.9 ± 2.62 a
	100	32.3 ± 0.24 a	1304.3 ± 170.83 a	2.7 ± 0.15 a	0.4 ± 0.03 a	72.9 ± 2.26 a
	200	33.6 ± 1.31 a	1064.0 ± 122.83 a	2.4 ± 0.22 ab	0.3 ± 0.04 a	68.1 ± 3.30 ab
Camellia japonica	Control	33.3 ± 1.60 a	27.5 ± 2.11 a	7.3 ± 0.28 a	3.7 ± 0.17 a	74.9 ± 1.90 a
	10	36.6 ± 3.43 a	25.8 ± 2.21 a	7.0 ± 0.54 a	4.0 ± 0.53 a	68.4 ± 5.89 a
	50	36.9 ± 2.13 a	19.8 ± 1.49 a	7.8 ± 1.07 a	4.2 ± 0.31 a	70.5 ± 5.28 a
	100	41.6 ± 3.16 a	21.5 ± 1.89 a	8.4 ± 0.21 a	4.3 ± 0.20 a	69.8 ± 3.94 a
	200	37.6 ± 3.22 a	24.8 ± 4.66 a	8.1 ± 0.42 a	4.1 ± 0.15 a	63.1 ± 5.09 a
Ternstroemia gymnanthera	Control	23.2 ± 1.12 b	28.7 ± 5.81 a	8.3 ± 0.25 a	3.6 ± 0.10 ab	40.7 ± 0.26 a
	10	33.3 ± 1.57 a	28.0 ± 7.81 a	7.7 ± 0.29 ab	3.8 ± 0.30 a	44.5 ± 3.96 a
	50	31.4 ± 3.33 a	36.0 ± 6.93 a	5.5 ± 0.85 c	2.4 ± 0.13 c	44.6 ± 6.81 a
	100	29.6 ± 3.47 ab	50.7 ± 4.06 a	6.1 ± 0.44 bc	2.8 ± 0.50 bc	41.6 ± 0.73 a
	200	32.2 ± 1.27 a	44.3 ± 8.35 a	5.8 ± 0.45 c	3.0 ± 0.12 a-c	37.2 ± 5.27 a

Farfugium japonicum	Control	22.9 ± 0.93 a	6.0 ± 0.58 a	26.3 ± 2.25 a	14.6 ± 0.32 ab	45.2 ± 1.22 a
	10	14.1 ± 0.83 b	5.0 ± 0.00 a	20.7 ± 1.20 b	13.5 ± 0.73 b	44.1 ± 1.75 a
	50	9.6 ± 1.51 c	6.0 ± 0.91 a	16.2 ± 0.39 c	15.3 ± 0.33 a	42.6 ± 0.49 a
	100	8.8 ± 0.71 c	4.3 ± 0.25 a	15.2 ± 0.63 c	14.2 ± 0.58 ab	46.3 ± 0.73 a
	200	9.5 ± 0.93 c	5.7 ± 0.88 a	15.6 ± 0.44 c	14.3 ± 0.43 ab	43.1 ± 4.15 a
Acorus gramineus	Control	-	24.0 ± 4.93 a	28.6 ± 2.39 a	0.7 ± 0.06 a	56.7 ± 3.46 a
	10	-	8.8 ± 1.16 c	22.6 ± 1.08 b	0.6 ± 0.04 b	54.6 ± 2.83 a
	50	-	12.4 ± 0.81 bc	23.6 ± 0.96 b	0.5 ± 0.02 b	55.6 ± 3.41 a
	100	-	16.2 ± 1.59 b	22.4 ± 0.75 b	0.5 ± 0.03 b	64.1 ± 3.87 a
	200	-	11.2 ± 2.08 bc	24.8 ± 1.60 ab	0.5 ± 0.04 b	60.5 ± 4.32 a
Liriope muscari	Control	-	48.3 ± 6.84 a[z]	32.4 ± 4.13 a	0.5 ± 0.03 a	36.6 ± 0.60 b
	10	-	38.3 ± 1.03 a	36.1 ± 2.65 a	0.7 ± 0.16 a	51.1 ± 1.86 a
	50	-	41.5 ± 3.71 a	33.0 ± 2.68 a	0.4 ± 0.05 a	55.8 ± 3.84 a
	100	-	39.3 ± 2.33 a	34.4 ± 3.32 a	0.4 ± 0.05 a	53.7 ± 2.92 a
	200	-	41.8 ± 4.05 a	34.5 ± 1.88 a	0.5 ± 0.03 a	55.0 ± 0.71 a

[1] Different letters indicate a significant difference using Duncan's multiple range test at $P < 0.05$. [2] Plants grown in a glass greenhouse.

Table S2. Effects of light intensity on chlorophyll fluorescence parameters of 19 plant species.

Species	Light intensity (PPFD)	Fv/Fm	Pi_Abs	ETo/RC	DIo/RC
Ilex × wandoensis	Control[1]	0.84 ± 0.01 a[2]	7.21 ± 1.48 bc	0.65 ± 0.10 a	0.21 ± 0.04 a
	10	0.84 ± 0.00 a	8.57 ± 0.18 ab	0.74 ± 0.03 a	0.21 ± 0.01 a
	50	0.85 ± 0.00 a	12.03 ± 1.87 a	0.72 ± 0.03 a	0.18 ± 0.00 a
	100	0.83 ± 0.02 a	10.54 ± 1.04 ab	0.68 ± 0.08 a	0.24 ± 0.08 a
	200	0.80 ± 0.02 a	4.10 ± 0.04 c	0.71 ± 0.05 a	0.30 ± 0.04 a
Euonymus japonicus	Control	0.73 ± 0.00 d	1.80 ± 0.10 b	0.92 ± 0.06 a	0.57 ± 0.02 a
	10	0.78 ± 0.00 a	3.13 ± 0.18 a	0.96 ± 0.03 a	0.42 ± 0.03 b
	50	0.77 ± 0.01 ab	2.24 ± 0.57 ab	0.90 ± 0.01 a	0.49 ± 0.05 ab
	100	0.76 ± 0.00 bc	2.55 ± 0.29 ab	0.83 ± 0.03 ab	0.45 ± 0.03 b
	200	0.74 ± 0.01 cd	1.99 ± 0.44 ab	0.75 ± 0.04 b	0.48 ± 0.02 ab
Cephalotaxus harringtonii	Control	0.58 ± 0.07 b	0.36 ± 0.28 b	0.57 ± 0.21 a	1.46 ± 0.44 a
	10	0.77 ± 0.01 a	1.99 ± 0.28 a	0.85 ± 0.03 a	0.47 ± 0.04 b
	50	0.77 ± 0.01 a	2.53 ± 0.71 a	0.83 ± 0.00 a	0.44 ± 0.05 b
	100	0.77 ± 0.00 a	2.28 ± 0.08 a	0.82 ± 0.03 a	0.44 ± 0.01 b
	200	0.75 ± 0.00 a	1.31 ± 0.26 ab	0.71 ± 0.02 a	0.51 ± 0.03 b
Rhododendron brachycarpum	Control	0.84 ± 0.01 a	6.98 ± 2.64 a	0.64 ± 0.06 a	0.21 ± 0.05 b
	10	0.82 ± 0.00 a	6.31 ± 0.41 a	0.69 ± 0.06 a	0.23 ± 0.01 b
	50	0.82 ± 0.00 a	7.35 ± 0.48 a	0.71 ± 0.04 a	0.23 ± 0.02 b
	100	0.73 ± 0.05 b	0.69 ± 0.34 b	0.75 ± 0.06 a	0.55 ± 0.13 a
	200	0.75 ± 0.01 b	2.05 ± 0.28 b	0.76 ± 0.06 a	0.46 ± 0.05 a
Quercus acuta	Control	0.69 ± 0.01 b	0.87 ± 0.04 b	1.00 ± 0.07 a	0.88 ± 0.08 a
	10	0.77 ± 0.01 a	2.42 ± 0.57 a	0.90 ± 0.04 ab	0.47 ± 0.05 b
	50	0.75 ± 0.01 a	1.47 ± 0.20 ab	0.92 ± 0.03 ab	0.59 ± 0.06 ab

	100	0.74 ± 0.01 a	1.41 ± 0.25 b	0.86 ± 0.03 ab	0.58 ± 0.04 ab
	200	0.69 ± 0.03 b	0.68 ± 0.16 b	0.76 ± 0.08 b	0.83 ± 0.18 a
Neolitsea sericea	Control	0.57 ± 0.09 b	0.35 ± 0.16 b	0.71 ± 0.13 a	1.92 ± 0.94 a
	10	0.74 ± 0.01 a	1.63 ± 0.15 a	0.89 ± 0.05 a	0.57 ± 0.05 a
	50	0.73 ± 0.01 a	1.35 ± 0.15 a	0.92 ± 0.04 a	0.63 ± 0.06 a
	100	0.67 ± 0.03 ab	0.62 ± 0.20 b	0.80 ± 0.04 a	0.92 ± 0.13 a
	200	0.57 ± 0.03 b	0.17 ± 0.02 b	0.70 ± 0.07 a	1.65 ± 0.24 a
Machilus thunbergii	Control	0.70 ± 0.03 ab	1.50 ± 0.59 a-c	1.03 ± 0.09 a	0.78 ± 0.14 a
	10	0.76 ± 0.01 a	2.19 ± 0.35 ab	0.88 ± 0.29 a	0.49 ± 0.05 b
	50	0.76 ± 0.00 a	2.29 ± 0.17 a	0.94 ± 0.01 a	0.50 ± 0.02 b
	100	0.71 ± 0.00 ab	1.11 ± 0.16 bc	0.88 ± 0.08 a	0.69 ± 0.02 ab
	200	0.67 ± 0.03 b	0.80 ± 0.17 c	0.84 ± 0.09 a	0.85 ± 0.11 a
Ardisia crispa	Control	0.83 ± 0.01 a	5.34 ± 0.01 a	0.64 ± 0.07 b	0.21 ± 0.01 b
	10	0.82 ± 0.00 a	5.73 ± 0.13 a	0.94 ± 0.02 a	0.31 ± 0.00 b
	50	0.82 ± 0.00 a	6.06 ± 0.36 a	0.89 ± 0.00 a	0.29 ± 0.00 b
	100	0.81 ± 0.01 a	4.90 ± 1.07 a	0.91 ± 0.04 a	0.34 ± 0.04 ab
	200	0.77 ± 0.02 b	4.02 ± 0.27 a	0.95 ± 0.02 a	0.47 ± 0.09 a
A. pusilla	Control	0.81 ± 0.02 a	3.02 ± 0.84 ab	0.67 ± 0.07 b	0.31 ± 0.04 b
	10	0.78 ± 0.00 ab	2.49 ± 0.15 b	0.82 ± 0.01 a	0.40 ± 0.01 a
	50	0.81 ± 0.00 a	4.33 ± 0.20 a	0.85 ± 0.02 a	0.31 ± 0.01 b
	100	0.79 ± 0.00 ab	3.02 ± 0.13 ab	0.90 ± 0.05 a	0.39 ± 0.01 a
	200	0.78 ± 0.01 b	2.42 ± 0.17 b	0.86 ± 0.05 a	0.45 ± 0.03 a
A. japonica	Control	0.79 ± 0.04 a	4.74 ± 1.72 a	0.49 ± 0.08 d	0.31 ± 0.09 a
	10	0.81 ± 0.00 a	5.00 ± 0.26 a	0.79 ± 0.01 bc	0.29 ± 0.00 a
	50	0.79 ± 0.00 a	4.05 ± 0.37 a	0.87 ± 0.03 ab	0.36 ± 0.01 a
	100	0.79 ± 0.00 a	3.88 ± 0.15 a	0.94 ± 0.02 a	0.39 ± 0.02 a
	200	0.81 ± 0.00 a	4.98 ± 0.72 a	0.72 ± 0.05 c	0.28 ± 0.01 a
Pittosporum tobira	Control	0.66 ± 0.00 a	0.40 ± 0.07 b	0.71 ± 0.07 a	0.99 ± 0.02 b
	10	0.37 ± 0.17 b	0.23 ± 0.21 b	0.38 ± 0.48 a	2.53 ± 1.54 a
	50	0.75 ± 0.00 a	1.65 ± 0.05 a	0.89 ± 0.06 a	0.55 ± 0.02 b
	100	0.74 ± 0.01 a	1.29 ± 0.13 a	0.87 ± 0.02 a	0.60 ± 0.04 b
	200	0.69 ± 0.02 a	0.63 ± 0.19 b	0.79 ± 0.06 a	0.86 ± 0.12 b
Rhaphiolepis indica var. umbellata	Control	0.73 ± 0.02 a	2.12 ± 0.39 a	0.81 ± 0.05 a-c	0.51 ± 0.04 a
	10	0.73 ± 0.02 a	1.89 ± 0.42 a	0.96 ± 0.03 a	0.61 ± 0.09 a
	50	0.75 ± 0.02 a	2.93 ± 0.81 a	0.88 ± 0.03 ab	0.49 ± 0.10 a
	100	0.75 ± 0.01 a	2.61 ± 0.53 a	0.78 ± 0.02 bc	0.45 ± 0.04 a
	200	0.73 ± 0.01 a	1.64 ± 0.52 a	0.67 ± 0.08 c	0.51 ± 0.06 a
Eriobotrya japonica	Control	0.61 ± 0.03 b	0.18 ± 0.23 d	0.11 ± 0.35 b	1.10 ± 0.15 a
	10	0.77 ± 0.00 a	2.33 ± 0.22 ab	1.05 ± 0.01 a	0.52 ± 0.01 c
	50	0.76 ± 0.01 a	2.51 ± 0.56 a	0.88 ± 0.05 a	0.47 ± 0.05 c
	100	0.73 ± 0.01 a	1.37 ± 0.41 bc	0.85 ± 0.08 a	0.64 ± 0.07 bc
	200	0.66 ± 0.02 b	0.56 ± 0.07 cd	0.73 ± 0.05 a	0.88 ± 0.05 ab
Torreya nucifera	Control	0.60 ± 0.01 b	0.54 ± 0.05 c	0.73 ± 0.01 bc	1.01 ± 0.04 a
	10	0.72 ± 0.03 a	1.90 ± 0.63 a-c	0.89 ± 0.10 a	0.65 ± 0.20 b
	50	0.76 ± 0.01 a	2.54 ± 0.66 a	0.84 ± 0.03 ab	0.47 ± 0.07 b
	100	0.75 ± 0.00 a	2.26 ± 0.17 ab	0.70 ± 0.02 bc	0.42 ± 0.01 b
	200	0.71 ± 0.01 a	0.89 ± 0.23 bc	0.63 ± 0.01 c	0.62 ± 0.06 b
Camellia japonica	Control	0.69 ± 0.01 b	1.08 ± 0.09 b	0.87 ± 0.03 a	0.73 ± 0.01 a
	10	0.75 ± 0.01 a	2.19 ± 0.31 a	0.83 ± 0.01 ab	0.48 ± 0.03 b
	50	0.75 ± 0.01 a	2.24 ± 0.45 a	0.76 ± 0.04 b	0.45 ± 0.03 b
	100	0.73 ± 0.01 a	1.50 ± 0.42 ab	0.81 ± 0.02 ab	0.59 ± 0.06 b
	200	0.70 ± 0.01 b	0.86 ± 0.18 b	0.85 ± 0.03 ab	0.77 ± 0.05 a
Ternstroemia gymnanthera	Control	0.82 ± 0.01 a	4.42 ± 0.45 ab	0.81 ± 0.11 a	0.29 ± 0.05 a

	10	0.79 ± 0.03 a	5.48 ± 0.77 a	0.94 ± 0.11 a	0.29 ± 0.05 a
	50	0.80 ± 0.01 a	2.63 ± 0.43 c	0.98 ± 0.09 a	0.44 ± 0.08 a
	100	0.76 ± 0.03 a	2.25 ± 0.27 c	0.94 ± 0.04 a	0.48 ± 0.05 a
	200	0.82 ± 0.01 a	2.99 ± 0.17 bc	0.81 ± 0.07 a	0.33 ± 0.04 a
Farfugium japonicum	Control	0.84 ± 0.00 a	6.15 ± 0.32 b	0.79 ± 0.03 a	0.24 ± 0.00 b
	10	0.81 ± 0.01 b	4.59 ± 0.82 b	0.75 ± 0.06 a	0.33 ± 0.01 a
	50	0.85 ± 0.00 a	10.48 ± 1.05 a	0.71 ± 0.03 a	0.18 ± 0.01 c
	100	0.85 ± 0.00 a	10.84 ± 0.92 a	0.71 ± 0.02 a	0.18 ± 0.01 c
	200	0.85 ± 0.00 a	8.96 ± 1.00 a	0.74 ± 0.03 a	0.20 ± 0.01 c
Acorus gramineus	Control	0.84 ± 0.00 a	4.99 ± 0.41 a	0.78 ± 0.41 b	0.25 ± 0.01 a
	10	0.83 ± 0.01 a	4.64 ± 0.06 a	4.64 ± 0.06 ab	0.26 ± 0.02 a
	50	0.81 ± 0.02 a	6.00 ± 0.89 a	6.00 ± 0.89 a	0.30 ± 0.07 a
	100	0.83 ± 0.01 a	5.25 ± 0.37 a	5.25 ± 0.37 ab	0.27 ± 0.03 a
	200	0.81 ± 0.01 a	5.68 ± 0.30 a	0.92 ± 0.06 ab	0.28 ± 0.02 a
Liriope muscari	Control	0.83 ± 0.01 a	3.94 ± 0.18 b	0.84 ± 0.06 a	0.30 ± 0.01 ab
	10	0.81 ± 0.00 a	5.15 ± 0.53 ab	0.85 ± 0.02 a	0.29 ± 0.01 ab
	50	0.82 ± 0.00 a	6.67 ± 0.58 a	0.77 ± 0.04 a	0.25 ± 0.02 b
	100	0.80 ± 0.02 a	5.75 ± 0.60 a	0.80 ± 0.05 a	0.27 ± 0.03 ab
	200	0.81 ± 0.00 a	5.44 ± 0.44 ab	0.88 ± 0.02 a	0.31 ± 0.01 a

[1] Different letters indicate a significant difference using Duncan's multiple range test at $P < 0.05$. [2] Plants grown in a glass greenhouse.
